# DNA Interaction with Coordination Compounds of Cd(II)containing 1,10-Phenanthroline

**DOI:** 10.3390/ijms25031820

**Published:** 2024-02-02

**Authors:** Nina Kasyanenko, Eugeny Belyi, Irina Silanteva, Victor Demidov, Andrei Komolkin

**Affiliations:** 1 Faculty of Physics, Saint Petersburg State University, 199034 Saint Petersburg, Russia; st077287@student.spbu.ru (E.B.); i.silanteva@spbu.ru (I.S.); a.komolkin@spbu.ru (A.K.); 2 I.V. Grebenshchikov Institute of Silicate Chemistry, Russian Academy of Sciences, 194021 Saint Petersburg, Russia; vndemidov@mail.ru

**Keywords:** Cd(II) compounds, 1,10-phenanthroline ligands, DNA binding, intercalation, molecular dynamics

## Abstract

The experimental study of the DNA interaction with three cadmium coordination compounds [Cd(phen)_3_](CH_3_CO_2_)_2_, [Cd(phen)_2_(H_2_O)_2_](CH_3_CO_2_)_2_, and [Cd_2_(phen)_4_(H_2_O)_2_](CH_3_CO_2_)_4_ was carried out using spectrophotometry, viscosity, and dynamic light scattering methods. The role of the solution ionic strength (concentration of NaCl) was analyzed. All compounds can penetrate (fully or partly) to the major or minor DNA grooves. It was shown that, in addition to the important role of electrostatic interactions in the formation of the complex, intercalation of the 1,10-phenanthroline ligand occurs for compounds [Cd(phen)_2_(H_2_O)_2_](CH_3_CO_2_)_2_ and [Cd_2_(phen)_4_(H_2_O)_2_](CH_3_CO_2_)_4_. Compound [Cd(phen)_3_](CH_3_CO_2_)_2_ binds to DNA externally. The coordination bond between cadmium and DNA was formed in DNA complexes with [Cd_2_(phen)_4_(H_2_O)_2_](CH_3_CO_2_)_4_. Preliminary computer modeling of the DNA interaction with the compounds used was performed.

## 1. Introduction

Metal complexes with different ligands can be used in medicine as antitumor and antibacterial drugs. A systematic study of their interactions with nucleic acids for the detection of the molecular mechanism of their biological activity is of the utmost importance in developing new drugs. Indeed, the DNA molecule is the main target of their therapeutic action.

DNA’s interaction with different coordination compounds of metals is among the most current research since the discovery and clinical development of the well-known anticancer drug cisplatin [1,2]. However, many aspects of the biological activity of platinum coordination compounds have not yet been identified [3]. Indeed, the mechanism of action of cisplatin is still not yet fully elucidated [4]. This demonstrates the relevance of studying the interaction of any complexes of metals with DNA in vitro to understand the molecular basis of their biological activity associated with their effect on DNA in cells.

DNA’s interaction with metal complexes depends on the nature of the metal, as well as on the type and location of ligands in the coordination sphere of ions. The coordination of metal to DNA bases may be accompanied by electrostatic interactions and the non-covalent binding of ligands from the coordination sphere of the central ion with DNA groups. Different ligands provide additional opportunities for the compound to specifically influence DNA’s properties. One of the most promising among the ligands used in coordination chemistry is phenanthroline. It is known that the phenanthroline ligands can enhance the antiproliferative activity of Pd(II) and Pt(II) complexes. Complexes of Pd(II) with phenanthroline have good pharmacokinetic and pharmacodynamic properties that make this class of compounds remarkable inhibitors, even of resistant cell growth [5]. Simple 1,10-phenanthrolines as excellent N-donor bidentate chelating ligands have been widely used to construct such compounds because of their excellent coordinating ability. They have a large conjugated system and can easily show π-π interactions like stacking [6,7,8] and other types of noncovalent contacts. Phenanthroline, both as a free molecule and as a ligand coordinated to metal centers, can bind with DNA base pairs. The ability of phenanthroline and its derivatives to intercalate into double-stranded DNA has been noted [9,10,11].

The synthesis of different metal complexes with phenanthroline ligands and the study of their binding to the DNA molecule have been carried out [11,12,13,14,15,16,17]. Some metal ions play an essential role in different biochemical functions. Biogenic ions Zn^2+^, Cu^2+^, and Mn^2+^ are widely used for the synthesis of metal complexes for medicine due to their biocompatibility. Cadmium and its compounds show high toxicity [18]. It is known that cadmium ions when connected with sulfhydryl groups of enzymes may destroy their functions [19]. Cd(II) can replace Zn(II) in proteins [20]. Highly toxic cisplatin shows the most striking results in the treatment of various tumors. Cadmium accumulates in cancer cells, so it can be used in some methods of antitumor therapy. The possible antitumor activity of different compounds is usually tested in vitro by studying their binding to DNA.

In our research, we examined DNA’s interactions with three cadmium compounds containing 1,10-phenanthroline: [Cd(phen)_3_](CH_3_CO_2_)_2_, [Cd(phen)_2_(H_2_O)_2_](CH_3_CO_2_)_2_, and [Cd_2_(phen)_4_(H_2_O)_2_](CH_3_CO_2_)_4_. The possibility of cadmium coordinating with DNA bases, the emergence of the stacking interaction of 1,10-phenanthroline ligands, and the eventuality of 1,10-phenanthroline intercalation between DNA bases during a complex formation were considered. To monitor the state of the secondary and tertiary structure of the DNA molecule during its interaction with cadmium compounds, methods of spectrophotometry, low-gradient viscometry, and dynamic light scattering were used. A preliminary computer simulation of DNA’s interactions with the compounds used was performed.

## 2. Results and Discussion

All compounds under study are acetates: [Cd(phen)_3_](CH_3_CO_2_)_2_, denoted as Cd(Phen)3; [Cd(phen)_2_(H_2_O)_2_](CH_3_CO_2_)_2_, denoted as Cd(Phen)2; and [Cd_2_(phen)_4_(H_2_O)_2_](CH_3_CO_2_)_4_, denoted as 2Cd(Phen)4.

When cadmium compounds Cd(Phen)3, Cd(phen)2, and 2Cd(Phen)4 are dissolved in water, complex ions with a charge of 2+ appear as a result of dissociation. The positive charge ensures the electrostatic attraction of cadmium ions to the negatively charged phosphate groups of DNA. The role of electrostatic interactions in the binding of cadmium compounds to DNA can be revealed by comparing the results of complex formation in solutions of low (0.005 M NaCl) and high (1 M NaCl) ionic strengths. An excess of counterions in 1 M NaCl provides the effective screening of the charge of the macromolecule.

**Figure 1:**
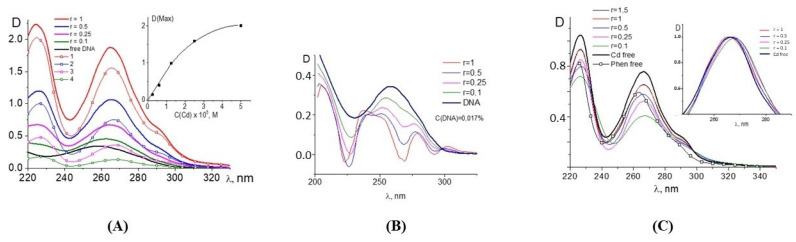
The results of the spectral analysis of a DNA interaction with Cd(Phen)3: the absorption spectra of DNA complexes with Cd(Phen)3 at constant DNA and different Cd concentrations (lines) and spectra of free compounds of the same concentrations: C(Cd) = 2.5 × 10^−5^ M (1), 1.25 × 10^−5^ M (2), 0.5 × 10^−5^ M (3), 0.25 × 10^−5^ M (4)—lines with signs; insert shows the dependence of the amplitude of the central band in the absorption spectra of a free compound on C(Cd), and the r value indicates the relation between C(Cd) and the molar concentration of DNA base pairs (**A**); the calculated absorption spectra of DNA in complexes with different r values at C(DNA) = 0.0017% = const. were obtained by the subtraction of the absorbance of free Cd(Phen)3 at the same concentrations as in complexes (**B**); calculated spectra of Cd(Phen) in complexes at 1.25 × 10^−5^ M = const. and different DNA concentrations obtained by the subtraction of the absorbance of free DNA the same concentrations as in complexes; insert shows normalized spectra (**C**).

Figure 1 demonstrates the spectral properties of the components during a DNA interaction with Cd(Phen)3 in 0.005 M NaCl. Unfortunately, the absorption spectrum of 1,10-phenanthroline ligands, which are responsible for the spectral properties of the cadmium compound, lies in the same frequency range as the analyzed DNA absorption band. This circumstance makes it difficult to analyze the binding using spectrophotometry. One can see two well-resolved bands with a maximum at 226 nm and 266 nm in absorption spectra of Cd(Phen)3 and one unresolved band in the wavelength region of 280–310 nm. In the spectrum of free 1,10-phenanthroline (Figure 1C), one can see two bands, shifted to the short-wavelength region relative to the absorption bands of Cd(Phen)3. The unresolved band in the region above 280 nm is practically not expressed.

The linear dependence of the intensity of the middle band of Cd(Phen)3 on the concentration of the compound (Figure 1A, inset) is disrupted at concentrations of more than 2 × 10^−5^ M. This may indicate a hypochromic effect due to the formation of associates with the stacking structures of 1,10-phenanthroline ligands. At lower concentrations, the absence of associates of the compounds can be manifested. Note that the positive charge of the complex ions does not contribute to the formation of associates. Apparently, an increase in the concentration of the compound in solution promotes the formation of such associates when 1,10-phenanthroline ligands of different complex ions form dimer structures. The comparison of the absorption spectrum of the compound Cd(Phen)3, containing three 1,10-phenanthroline ligands, with the absorption spectrum of a 1,10-phenanthroline with the same concentration of chromophores shows a greater hypochromic effect for free 1,10-phenanthrolines than for 1,10-phenanthrolines in the coordination sphere of the compounds. Thus, in solutions of free 1,10-phenanthroline, the formation of dimers occurs more easily than in solutions of Cd(Phen)3.

Figure 1A shows the absorption spectra of DNA complexes with Cd(Phen)3 in 0.005 M NaCl when the DNA solution was added into Cd(Phen)3 solutions of different concentrations. C(DNA) in complexes was 0.0017%. The r value gives the ratio of the Cd concentration in the solution to the DNA base-pair molar concentration. We subtracted the absorbance of free Cd(Phen)3 and calculated the DNA absorption in the complexes (Figure 1B) with the assumption that the spectral properties of the cadmium compound remained unchanged during the complex formation (which is incorrect). Indeed, the obtained DNA spectra demonstrate an unrealistic shape. Such spectra may be obtained due to a decrease in the absorption of Cd(Phen)3 in the complexes. The hypochromic effect occurs in two bands, and this can produce the observed result.

The results of another experiment with the same concentration of Cd(Phen)3 and different DNA concentrations also clearly demonstrate a hypochromic effect in the spectrum of Cd(Phen)3 upon binding (see calculated spectra of Cd(Phen)3 in complexes with DNA in Figure 1C). Naturally, in such a calculation, we assume that the absorption of DNA in a complex coincides with that of free DNA. The hypochromic effect is most noticeable in solutions with high DNA concentrations (small r value) and with an excess of binding sites for the compounds on DNA. We believe that, in this case, the appearance of stacking interactions of chromophores in the complexes can be observed. This may be either the result of the intercalation of 1,10-phenanthroline between DNA bases or the formation of a complex when different 1,10-phenanthroline ligands form stacked associates. Note that free 1,10-phenanthroline has another spectral band (see Figure 1C). A small shift in the absorption band of Cd(Phen)3 with a decreasing r value also indicates the binding and changes in Cd(Phen)3 spectra in the complexes.

**Figure 2:**
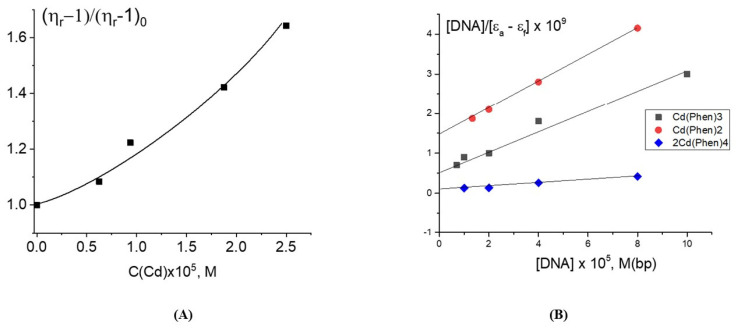
The dependence of the relative change in the reduced viscosity of DNA solutions on the Cd(Phen)3 concentration at C(DNA) = 0.0033% = 5 × 10^−5^ M in 0.005 M NaCl (**A**), and a Wolfe-Shimer plot to determine the binding constant for three cadmium compounds in their interactions with DNA in 0.005 M NaCl (**B**).

The increase in the reduced viscosity of DNA solutions at a constant DNA concentration is observed with the growth of the Cd(Phen)3 concentration (Figure 2A) at low ionic strength (0.005 M NaCl). This result may be explained by several reasons: the intercalation of the 1,10-phenanthroline ligands of the compound between DNA bases, an increase in the persistent length of DNA during the formation of its complexes with cadmium compounds, and the appearance of intermolecular DNA–DNA aggregates. The relation between the intrinsic viscosity of DNA [η] and the parameters of the macromolecule is given by the Flory formula:

$$\left[\eta \right]=\;\Phi \frac{{<h^{2}>}^{3/2}}{M}=\;\Phi \frac{{<{h^{2}}_{0}>}^{3/2}}{M}\alpha^{3}=\;\Phi \frac{{\left(LA\right)}^{3/2}}{M}\alpha^{3}$$

where Ф is a Flory parameter, M is the DNA molecular mass, *L* is the hydrodynamic length of the polymer chain, and *A* is the length of the Kuhn segment (chain rigidity parameter).

For high molecular DNA samples, A = 2p, where p is the persistent length, <h^2^>^1/2^ and <h_0_^2^>^1/2^ are the mean-square distances between the ends of the DNA chain in the real and in the ideal solutions, and α is the coefficient of linear swelling, α = <h^2^>^1/2^/<h_0_^2^>^1/2^. The intrinsic viscosity [*η*] of DNA is found by extrapolating the reduced viscosity to a zero DNA concentration at zero gradient:

$$\left[\eta \right]={lim}_{\begin{array}{c} C\to 0\\ g\to 0 \end{array}}\frac{\left(\eta_{r}-1\right)}{C}$$

The relative change in the reduced viscosity of solutions $\eta_{red}=\left(\frac{\eta_{r}-1}{C}\right)$ at the same DNA concentration may reflect the change in the DNA volume *V* ~ <h_0_^2^>^3/2^ α^3^ = (2*Lp*)^3/2^ α^3^.

Since the compound Cd(Phen)3 has a positive charge, it is natural to assume its electrostatic attraction to the negatively charged DNA. However, electrostatic binding should cause a drop in the solution viscosity due to the shielding of the DNA charges. The existence of other types of binding must be regarded (this does not exclude the electrostatic interaction). One of the popular models of the binding of metal complexes with 1,10-phenanthroline ligands to DNA is intercalation or the insertion of 1,10-phenqnthroline between the planar bases of deoxyribonucleic acid. For ligands during intercalation, specific changes in their absorption spectra along with an increase in the viscosity of DNA solutions must be observed [21,22].

The intercalation of ligands stabilizes the double-stranded structure. DNA intercalators can be cationic or neutral. They cause DNA helix unwinding and helix elongation (increase in *L* value). The unwinding of a DNA helix causes the base pairs to separate, creating an opening of about 0.34 nm (3.4 Å). This type of binding causes changes in the spectral properties of ligands, such as DNA-induced hypochromism and a shift of the absorption band. DNA lengthening and unwinding usually are determined from the change in the viscosity of a solution of linear DNA upon the addition of ligands. Hydrodynamic measurements are very sensitive to length changes in DNA. The significant increase in the viscosity of the DNA solution with complexes is obviously due to the increase in the overall DNA contour length *L* [23]. The intercalation binding obeys the rule of nearest-neighbor exclusion [24]. Indeed, the intercalating ligand can be inserted in such a way that the neighboring DNA base pair does not participate in the intercalation. Hence, we can conclude that the increase in the intrinsic viscosity of DNA due to the elongation of the macromolecule during intercalation at the maximum possible binding without a change in the DNA rigidity according to the Flory formula should not exceed 80%. For the reduced viscosity of DNA solutions, this change may be different.

Figure 2A shows a large increase in the DNA solution viscosity with a growth of the Cd(Phen)3 concentration. The intercalation of the 1,10-phenanthroline ligands of the compound Cd(Phen)3 between the bases of the macromolecule is sterically hindered: it is inconvenient due to the specific orientation of 1,10-phenanthrolines in the coordination sphere of the central ion. This becomes possible only as a result of the release of 1,10-phenanthroline from the coordination sphere of the cadmium. At the same time, an increase in the persistent length of DNA during binding also cannot explain the data obtained. Indeed, the increase in viscosity is too high. Based on the data reviewed, we also cannot unambiguously confirm or refute the assumption about intercalation. But the formation of DNA–DNA aggregates can be confirmed experimentally. The viscosity of the DNA solution in this case increases, and its dependence on the Cd(Phen) concentration is non-linear.

Estimation of the binding constant of Cd(Phen)3 in 0.005 M NaCl using the Wolfe-Shimer plot (Figure 2B) gave K_b_ = (5.0 ± 1.0) × 10^4^ M^−1^.

This value indicates the absence of coordination bonds between cadmium and DNA. Possibly as a result of the electrostatic attraction of positively charged complex ions to negatively charged DNA phosphates, a complex formation occurs, which can be classified as external binding. However, the results of the experiment discussed above indicate a change in the spectral properties of the 1,10-phenanthroline ligands of the compound upon the binding. Compensation of the charge of a compound upon binding to DNA can provoke the association of hydrophobic 1,10-phenanthroline ligands, attracting complex ions to each other, including those bound to distant DNA sites or even bound to another DNA molecule. As a result, Cd(Phen)3-Cd(Phen)3 associates with dimers of 1,10-phenanthroline, and the intermolecular DNA–DNA aggregates can be formed. Indeed, for DNA connected to externally bound complex ions containing hydrophobic ligands, the polymer–solvent affinity changes significantly, which promotes the formation of intermolecular contacts.

**Figure 3:**
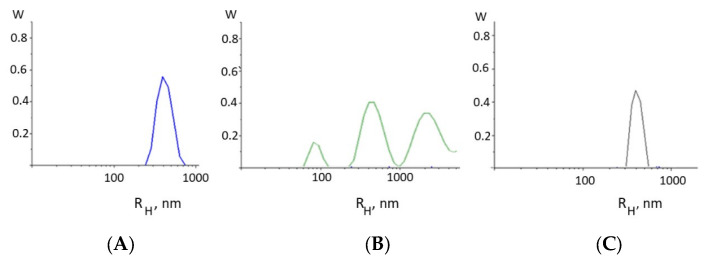
The size distribution function of the particles in a DNA solution without (**A**) and with cadmium compounds: Cd(phen)3 at C(Cd) = 4.5 × 10^−5^ M (**B**) and Cd(Phen)2 at C(Cd) = 10^−5^ M (**C**) in 0.005 M NaCl at a scattering angle 90 °C (DNA) = 0.002%.

The formation of intermolecular DNA–DNA bonds is evidenced, for example, from the results of the dynamic light scattering method (Figure 3). Indeed, the appearance of intermolecular DNA–DNA associates in the solution as a result of DNA complexation with Cd(Phen)3 molecules follows from the type of the particle size distribution function obtained from DLS measurements (Figure 3B). For DNA (Figure 3A), the results demonstrate the presence in the solution of particles with a hydrodynamic radius of 350 ± 50 nm, which corresponds to the mode responsible for the translational motion of the macromolecule as a whole.

We can estimate the gyration radius of the DNA used with a molecular mass of 11 × 10^6^ g/mol as <*R_g_*^2^>^1/2^ = 310 nm. Indeed, the hydrodynamic (contour) length of DNA is *L* = 5700 nm. The root-mean-square end-to-end distance of the chain in an ideal solution is <*h*_0_^2^> = *LA*, and the radius of gyration is <*R_g_*^2^> = (1/6) <*h*_0_^2^>. However, in 0.005 M NaCl, the size of the macromolecule will increase as a result of polyelectrolyte swelling, but due to the high rigidity of DNA, the linear swelling coefficient in this case is small. In principle, the value of the gyration radius obtained using the DLS method quite adequately reflects the actual size of a molecular coil under this condition.

For a DNA complex with Cd(Phen)3, this mode shifts and corresponds to particles with a hydrodynamic radius of 450 ± 50 nm. This agrees well with the viscometry result. The small mode in this case may be attributed to an association of free cadmium compounds in a solution. The data also indicate the emergence of large particles like intermolecular DNA aggregates in the solution.

The tendency to form aggregates in DNA solutions with Cd(Phen)3 is also evidenced from the results of examining systems in 1 M NaCl, with the excess salt. DNA solutions with a high enough concentration of Cd(Phen)3 in this case are unstable. The turbidity in the solutions indicates a phase separation. No turbidity was observed at low concentrations of the compound in DNA solutions or in solutions without DNA.

The analysis of all experimental data obtained for DNA complexes with Cd(Phen)3 indicates that complexes were formed with the participation of the 1,10-phenanthroline ligands of the compound, which remain part of the cadmium coordination sphere (the spectrum of free 1,10-phenanthroline differs significantly). The hypochromic effect in the absorption spectrum of the compound during its binding to DNA shows the formation of stack-like associates of 1,10-phenanthrolines, although this is sterically inconvenient for Cd(Phen)3. This can occur outside the DNA helix between ligands belonging to two neighboring complex ions bound to the macromolecule. The insertion of 1,10-phenanthroline ligands between heterocyclic DNA bases (intercalation model) is possible but unlikely. The value of the binding constant indicates the absence of a coordination bond between cadmium and DNA during the interaction.

**Figure 4:**
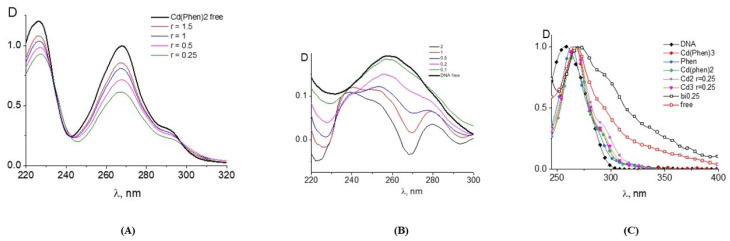
The results of the processing of the absorption spectra of the DNA complexes with Cd(Phen)2 in 0.005 M NaCl: the calculated spectra of Cd(Phen)2 in complexes at C(Cd) = 2 × 10^−5^ M = const. (**A**); the calculated DNA absorption spectra in complexes at constant DNA concentration; the r value is given near the corresponding lines (**B**). Calculations were performed in the same way as indicated above for the spectra presented in Figure 1. (**C**) shows the calculated and normalized values on the maximum of the band spectra of the free compounds and compounds of Cd(Phen)3, Cd(Phen)2, and 2Cd(Phen)4 in complexes with DNA.

The data obtained for DNA complexes with the compound Cd(Phen)2 with two 1,10-phenanthroline ligands are presented in Figure 4. Two positions in the coordination sphere of the cadmium ion are occupied by water molecules, which can be replaced by incoming DNA groups. The absorption spectra of DNA complexes with Cd(Phen)2 in 0.005 M NaCl are similar to those observed for DNA-Cd(Phen)3 complexes. The result of their processing also shows the hypochromic effect in spectra of a cadmium compound after its binding to DNA. This is evident both from the drop in absorption for the calculated spectra of the compound in complexes and from the form of the calculated DNA spectra in complexes (Figure 4A,B). It follows that, upon binding, the spectral properties of phenanthroline ligands change almost identically for both compounds. At the same time, spectral changes in complexes are greater for Cd(Phen)2 compared to Cd(Phen)3 (Figure 4C). Note that in the absorption spectra of flat ligands that form stacking structures upon external binding to DNA, a hypochromic effect with no shift was observed [25]. This is typical for complexes of the compound Cd(Phen)3 with DNA.

**Figure 5:**
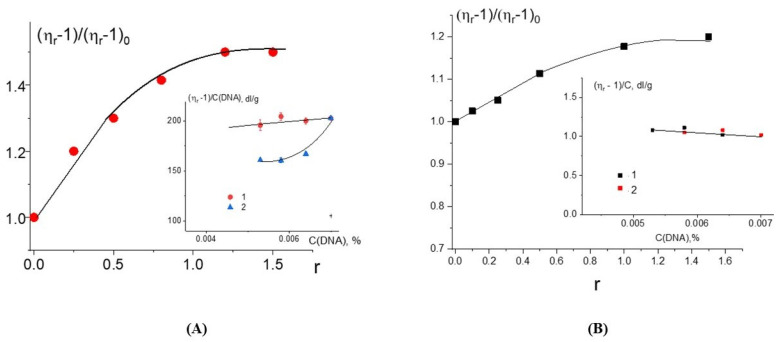
The dependence of the relative change in the reduced viscosity of DNA solutions on r in 0.005 M NaCl for Cd(Phen)2 (**A**) and 2Cd(Phen)4 (**B**) in 0.005 M NaCl. The insets in (**A**) and in (**B**) show the results of different dilutions of the DNA complex with corresponding compounds of cadmium compounds: 1—dilution with 0.005 M NaCl, 2—dilution with same C(Cd) like in initial complexes). The size of the dots shows the measurement error.

The growth of the relative change in the reduced viscosity of DNA solutions with the increasing r value at a constant DNA concentration for DNA complexes with Cd(Phen)2 (Figure 5A) may be associated primarily with intercalation. But we cannot exclude another reason (increase in the persistent length of DNA, for example) for the growth of the viscosity. At Cd(Phen)2 concentrations more than 5 × 10^−5^ M (at r > 1), the increase in viscosity stops. It is possible that the saturation of one of the types of binding is observed. This to some extent argues against the formation of DNA aggregates in solution, since in this case, it is impossible for the viscosity to remain unchanged with an increasing concentration of the compound that provokes the formation of such aggregates. This conclusion is also supported by the data of the DLS method. We cannot exclude the emergence of intermolecular aggregates, but Figure 3C did not show the presence of large particles in the DNA solution at the concentration used. Apparently, when binding, the Cd(Phen)2 compound is not located on the surface of the DNA but is located in its grooves.

The assessment of the binding constant for this compound to DNA (Figure 2B) gives K_b_ = (3 ± 1) × 10^5^ M^−1^, which is almost an order of magnitude greater than for Cd(Phen)3. The 1,10-phenanthroline ligands of the compound Cd(Phen)2 in complexes with DNA are in the cadmium-bound state (the absorption spectra of free 1,10-phenanthroline and 1,10-phenanthroline in complexes are shifted to shorter wavelengths). The simulation shows that Cd(Phen)2 has two structures: with cis- and trans-orientations of 1,10-phenanthrolines. The orientation of 1,10-phenanthroline in the coordination sphere of cadmium must be convenient for the intercalation if the complex ion is in a DNA groove due to the electrostatic attraction and good steric conditions.

Whether a coordination of a cadmium ion to DNA is observed for Cd(Phen)2 can be checked via the experiment with different dilutions of a pre-prepared complex (stock solution) of DNA with Cd(Phen)2 in 0.005 M NaCl. This stock solution was kept for 8 h before the study after preparing (mixing together of solutions with DNA and Cd(Phen)2) for the reliable formation of the coordination bonds. This solution was diluted to test the type of concentration dependence either with a NaCl solution (when C(Cd)/C(DNA) = constant) or with a solution of Cd(Phen)2 (in this case, C(Cd) = const). If strong nonequilibrium binding occurs in complexes (coordination bonds), both dilutions will lead to the same dependence of the reduced viscosity on the DNA concentration [16]. For the case when the equilibrium between the fraction of free and the fraction of bonded compounds changes with the decrease in DNA concentration, these two methods of dilution will lead to different concentration dependencies observed in our experiment (see insert in Figure 5A). Thus, the compound Cd(Phen)2 does not form a coordination bond with DNA. The intercalation model with the location of a compound in the minor or major groove of DNA between two negatively charged phosphates that neutralize the charge of the cadmium may be offered. It should be emphasized once again that, despite the great similarity in the spectral changes of Cd(Phen)2 and Cd(Phen)3 in complexes with DNA, the difference in binding constants suggests that a stronger binding is observed for Cd(Phen)2. Indeed, in addition to the electrostatic binding, the intercalation of 1,10-phenanthroline occurs.

**Figure 6:**
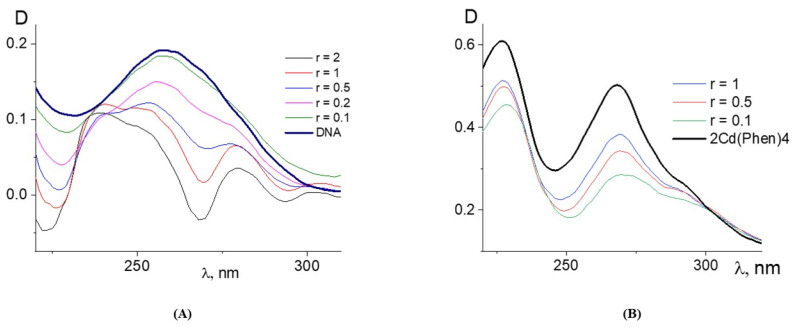
The results of the processing of the absorption spectra of DNA complexes with 2Cd(Phen)4 in 0.005 M NaCl: the calculated spectra of DNA in complexes at a constant DNA concentration C(DNA) = 0.001% (**A**) and the calculated absorption spectra of 2Cd(Phen)4 in complexes at a constant 2Cd(Phen)4 concentration C(Cd) = 0.5 × 10^−5^ M = const (**B**).

The third compound was the binuclear cadmium complex 2Cd(Phen)4. The internal coordination sphere of both cadmium ions in this compound is the same as in Cd(Phen)2. The processing of the absorption spectra of DNA complexes with the binuclear compound (Figure 6) indicates a great similarity with spectral changes observed for DNA-Cd(Phen)2 complexes. The Wolfe-Shimer plot for the binuclear compound gives a binding constant of K_b_ = (5 ± 1) × 10^5^ M^−1^. The experiment with two different methods of dilution of the initial DNA-2Cd(Phen)4 complex for the construction of the concentration dependence shows similar results typical for strong nonequilibrium binding (see insert in Figure 5B). The binding is stronger than observed for the Cd(Phen)2 interaction with DNA. We can assume the coordination binding of a compound to DNA with the intercalation of 1,10-phenanthroline. This is possible when a complex ion penetrates to the major groove of DNA. Then, the cadmium can coordinate with N7 guanine, and 1,10-phenanthroline can intercalate. DNA intercalation can involve the insertion of one intercalating moiety (mono-intercalator) and two intercalating moieties (bis-intercalator). From a comparison of the dependence of the viscosity of DNA solutions on r for compounds Cd(Phen)2 and 2Cd(Phen)4, it follows that only one of the two complex ions of the 2Cd(Phen)4 compound interacts with DNA. In addition, the presence of two independent types of binding (the formation of a coordination bond and the intercalation of 1,10-phenanthroline) can be assumed. The increase in viscosity of DNA solutions with 2Cd(Phen)4 is less than with Cd(Phen)2, but the saturation of binding is observed at the same r value.

**Figure 7:**
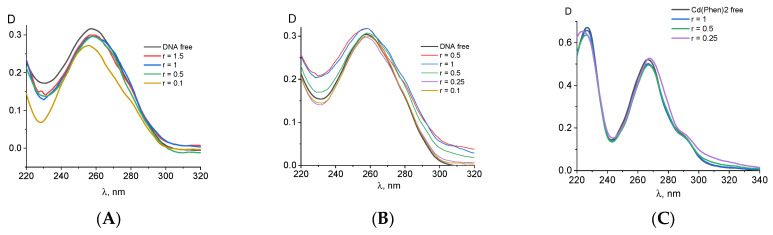
The results of the processing of the absorption spectra of DNA complexes with cadmium compounds in 1 M NaCl: calculated DNA absorption spectra in complexes with Cd(Phen)3 (**A**) and with 2Cd(Phen)4 (**B**) at C(DNA) = 0.0015%; calculated absorption spectra of Cd(Phen)2 in complexes at constant Cd(Phen)2 concentration C(Cd) = 1.5 × 10^−5^ M (**C**). Calculations were performed in the same way as indicated above.

The absorption spectra of DNA complexes with three cadmium compounds in 1 M NaCl show approximately the same results. In 1 M NaCl, the interaction of compounds with DNA is not so strong. Figure 7 shows some results of processing these spectra. The estimation of the binding constants for three compounds in 1 M NaCl gives values around 10^4^ M^−1^. This result indicates that the electrostatic interactions in DNA binding with compounds in 0.005 M NaCl are very important. In 1 M NaCl, the turbidity and precipitation at a high concentration of cadmium compounds in DNA solutions appeared over time. The solutions of DNA with 2Cd(Phen)4 show relative stability. We can conclude that for DNA complexes with cadmium compounds containing hydrophobic ligands, this solvent with a high salt concentration is thermodynamically poor. These results confirm the influence of hydrophobic ligands on intermolecular interactions in DNA solutions with the cadmium compounds used in 0.005 M NaCl.

### Simulation Results

Unlike the experiment results, the simulation shows that Cd(Phen)3 in water (system I) and in 1 M NaCl solution (system II) did not form associates under the conditions used. In a salt solution, they can approach each other at a distance of more than 4 Å (between their hydrogen atoms).

**Figure 8:**
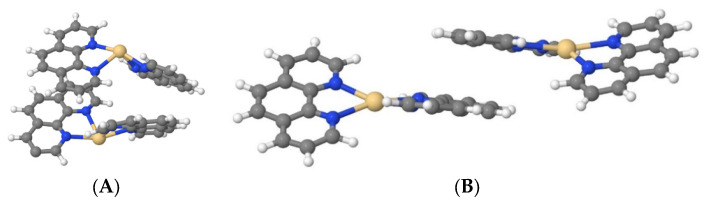
A stable dimer of cis-Cd(Phen)2 (**A**) and an unstable dimer of trans-Cd(Phen)2 in 1 M NaCl (**B**). Water molecules in the Cd coordination sphere are not shown.

Cd(Phen)2 in the cis-conformation in water (system III) also did not form associates; however, in a 1 M NaCl (system IV), the formation of two dimers among nine complex ions of Cd(Phen)2 was observed. At first, Cd(Phen)2 molecules stick together via 1,10-phenanthroline residue (the residues are parallel). This is an unstable state, which then transforms into a stable state with two 1,10-phenathroline residues opposite each other (Figure 8A). In this state, there are no water molecules between the Cd(Phen)2. When the salt ions were removed from system IV (system V), both dimers disintegrated. In the manually created system with a higher initial start concentration of Cd(Phen)2 in the form of dimers (system VI) in water for 30 ns, all dimers disintegrated too. So, the simulations show the impossibility of the existence of cis-Cd(Phen)2 dimers in unsalted solutions.

A total of 6 out of 18 dimers disintegrated in 100 ns in the manually created system with Cd(Phen)2 initially in the form of dimers in a solution of 0.15M NaCl (system VII); that is, at such a salt concentration, the existence of both dimers and monomers is possible. In 0.5 M NaCl (system VIII), 2 out of 18 dimers disintegrated in 50 ns, which means that in this salt concentration, Cd(Phen)2 exists predominantly in the form of dimers.

Cd(Phen)2 in the trans-conformation in water (system IX), as well as in the cis-conformation, does not form the dimers. In a 1 M NaCl solution (system X), the stable dimers are not formed, although unstable forms are possible (with an average lifetime no longer than 1 ns) when the trans-Cd(Phen)2 isomers cling together via the 1,10-phenanthroline residues with a parallel orientation (Figure 8B).

**Figure 9:**
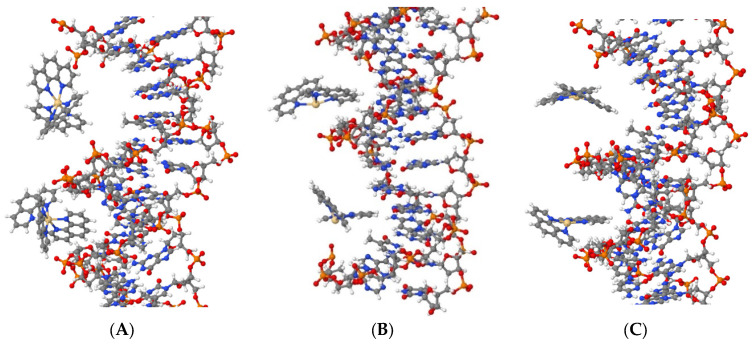
Cd(Phen)3 (system DI, 43 ns) (**A**), cis-Cd(Phen)2 (system DII, 6 ns) (**B**), and trans-Cd(Phen)2 (system DIII, 38 ns) (**C**) in major and in minor grooves of DNA. In (**A**,**C**), the major groove is at the top, and in (**B**), it is at the bottom. Water molecules in the Cd coordination sphere are not shown.

The simulation shows that all three compounds, Cd(Phen)3 (system DI), cis-Cd(Phen)2 (system DII), and trans-Cd(Phen)2 (systems DIII), are attracted to DNA phosphates due to their positive charge. In addition, they can penetrate (fully or partially) into the major and minor grooves of DNA (see Figure 9). Cd(Phen)3 predominantly enters the major groove, and the penetration into the minor group is partial, i.e., by one 1,10-phenanthroline residue. Cd(Phen)3 cannot enter the minor groove fully due to its large size. Cd(Phen)2 (in cis- and trans-conformations) enters predominantly into the minor groove of DNA. It can be noted that they are attracted to phosphates. Note that the simulation used cannot visualize the intercalation of the 1,10-phenanthroline ligand. At this stage, we can say that the task regarding the DNA interaction with the considered compounds requires a more detailed analysis, which we will carry out in further work.

## 3. Methods and Materials

### 3.1. Materials and Experimental Methods

Commercial samples of 1,10-phenanthroline monohydrate, phen·H_2_O (cleaned for analysis) and cadmium acetate dihydrate Cd(AcO)_2_·2H_2_O (chemically pure) were purchased from LenReactiv and NevaReactiv (Russia). Mononuclear complexes of Cd(II) with 1,10-phenanthroline Cd(Phen)3 and Cd(Phen)2 were obtained via complexation reactions of cadmium acetate Cd(CH_3_CO_2_)_2_•2H_2_O and 1,10-phenanthroline phen•H_2_O in an aqueous solution. The binuclear bridged complex 2Cd(Phen)4 was synthesized with a new methodology of metal-assisted non-dehydrogenative C(sp2)H coupling of 1,10-phenanthroline as part of d-element complexes [26].

For the synthesis of Cd(Phen)3, the crystalline cadmium acetate dihydrate Cd(CH_3_CO_2_)_2_•2H_2_O was added to the molten 1,10-phenanthroline monohydrate phen•H_2_O at a temperature of about 110–120 °C and stirred in portions in a molar ratio of 3:1. The melt was stirred for half an hour. After the almost colorless melt was cooled, the compound solidified in the form of a transparent glassy mass. An elemental analysis found (%) C 61.9, H 3.8, and N 10.7, and the calculations for C_40_H_30_N_6_O_4_Cd (%) yielded C 62.18, H 3.89, and N 10.88.

For the synthesis of Cd(Phen)2 to a concentrated aqueous solution of cadmium acetate dihydrate Cd(CH_3_CO_2_)_2_•2H_2_O 1.10-phenanthroline monohydrate phen•H_2_O was added in portions with stirring at a molar ratio of 1:2 and room temperature. The colorless solution was heated to 90–95 °C, and heating was continued for about 1 h. After evaporation of the water, the colorless compound crystallizes. The elemental analysis found (%) C 53.4, H 4.0, and N 8.8, and the calculations for C_28_H_26_N_4_O_6_Cd (%) yielded C 53.50, H 4.14, and N 8.92.

For the synthesis of 2Cd(Phen)4 or (Phen)Cd(µ-Phencyan)Cd(Phen)(CH_3_CO_2_)_4_ acetate bis(1,10-phenanthroline)(µ-1.10-phenanthrocyanine)dicadmium(II), a mixture of crystalline 1,10-phenanthroline monohydrate phen•H_2_O and crystalline cadmium acetate dihydrate Cd(CH_3_CO_2_)_2_•2H_2_O at a molar ratio of 2:1 was ground in a mortar and then heated to 110–120 °C to form a homogeneous colorless melt. The melt was kept at this temperature for about half an hour, and then the temperature was raised to 200–205 °C and again maintained at this temperature for about 25 min. The mass boiled and gradually changed its color from almost colorless to black and purple. The compound was formed in a glassy state of black and purple color. The elemental analysis found (%) C 55.9, H 3.4, and N 9.0, and the calculations for C_56_H_44_N_8_O_8_Cd_2_ (%) gave C 56.76, H 3.72, and N 9.46.

Some physicochemical methods were used to characterize DNA complexes with cadmium compounds. Spectrophotometry makes it possible to analyze the changes in the absorption spectra of components during the binding. The data obtained may be used to determine the binding constant of cadmium compounds to DNA with the Wolfe-Shimer approach [27].

$$\begin{array}{c} \frac{\left[DNA\right]}{\left|\varepsilon_{a}-\varepsilon_{f}\right|}=\frac{\left[DNA\right]}{\left|\varepsilon_{b}-\varepsilon_{f}\right|}+\frac{1}{K_{b}\left(\left|\varepsilon_{b}-\varepsilon_{f}\right|\right)} \end{array}$$

where *K_b_* is a binding constant, *ε_a_*, *ε_b_*, and *ε_f_* are coefficients of the molar extinction of the compound in the DNA solution at a certain concentration, for a completely bound compound and for free compounds in solution, respectively; and [DNA] is the DNA concentration. The dependence of [DNA]/|*ε_a_* − *ε_f_* | on [DNA] ensures the $K_{b}$ value is found.

The low-gradient rotational viscometer of the Zimm–Crothers type [28] was used to study the viscosity of DNA solutions during the binding with cadmium compounds. The relative change in the reduced viscosity of DNA solutions at constant DNA and different cadmium concentrations shows a change in the volume of the DNA molecular coil during the formation of complexes. The measurements were carried out at a temperature of 21 °C. The relative viscosity of DNA solutions *η_r_* was determined at flow velocity gradients less than 1.5 s^−1^, which made it possible to not study the gradient dependence of the *η_r_* value when calculating the reduced viscosity of the DNA solutions, ${\eta_{red}=(\eta }_{r}-1)/C$.

The analysis of the correlation functions of the scattered light intensity in the dynamic light scattering (DLS) method gives the translational diffusion coefficients of DNA molecules *D_t_* in complexes with cadmium compounds. We estimated the hydrodynamic radius of macromolecules R_H_ using the Stokes–Einstein formula for spherical particles:

$$D_{t}=\frac{k_{B}T}{6\pi \eta R_{h},}$$

*T* is the absolute temperature, *η* is the viscosity of the medium, and *k_B_* is the Boltzmann constant. The Photocor Complex and DynaLS software (Version 2.7.1) were used.

A commercial sample of high molecular weight calf thymus DNA from Sigma Aldrich was used in this research. The molecular weight of DNA 10^7^ g/mol was determined with viscometry. DNA solutions were used with the addition of a supporting electrolyte NaCl at concentrations of 0.005 M or 1 M.

### 3.2. Model and Simulation Methods

**Figure 10:**
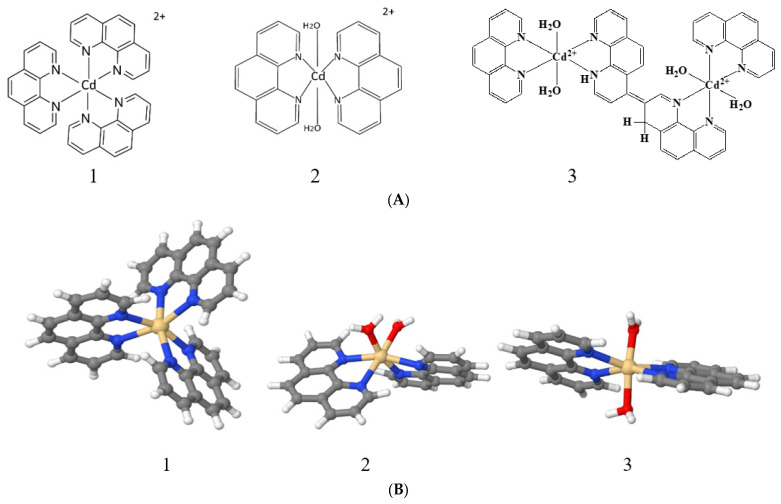
The chemical structure of the coordination sphere of complex ions (**A**) for compounds Cd(Phen)3 (1), Cd(phen)2 with two water molecules in the coordination sphere of ion (2), 2Cd(Phen)4 (3), and the models of Cd(Phen)3 (1) and Cd(Phen)2 in cis (2) and trans (3) conformations with the two nearest water molecules. (**A**) The chemical structure of the coordination sphere of complex ions. (**B**) Models of Cd(Phen)3 (1) and Cd(Phen)2 in cis (2) and trans (3) conformations.

The chemical structure of the coordination sphere of complex ions for compounds Cd(Phen)3, Cd(phen)2, 2Cd(Phen)4, and the models of compounds are presented in Figure 10.

The quantum chemical calculations of the structure of cadmium complexes with three phenanthroline ligands Cd(Phen)3 and with two phenanthroline ligands Cd(Phen)2 were carried out using the Sapporo-dZP-2012 basis. The structures of these compounds are presented in Figure 10B. Theoretically, for Cd(Phen)2, two structures with cis- and trans-conformations are allowed. The energy of the conformations was calculated using the Sapporo-TZP-2012+diffuse basis. The energy of cis-conformation (−6752.83366 hartree) turns out to be lower than the energy of trans-conformation (−6752.82841 hartree). We can assume that Cd(Phen)2 exists predominantly in the cis-conformation, although both conformations are regarded further.

The several systems containing Cd(Phen)3 or Cd(Phen)2 in water or in NaCl solution were simulated using molecular dynamics. The composition of all simulated boxes is given in Table 1.

System I consists of 9 complex ions of Cd(Phen)3 in water. System II also has Na^+^ and Cl^−^ ions at a 1 M concentration. System II is obtained from system I via the addition of Na^+^ and Cl^−^ ions after 60 ns of simulation time. Systems III and IV have 9 complex ions of cis-Cd(Phen)2 in water and in the presence of salt (Na^+^, Cl^−^ ions) at a 1 M concentration, respectively. System IV was obtained from system III via the addition of Na^+^ and Cl^−^ ions after 16 ns of simulation time. System V was obtained from system IV after completing the simulation of system IV by removing Na^+^ and Cl^−^ ions. Systems containing 36 complex ions of cis-Cd(Phen)2 in the form of dimers in water (system VI) and in 0.15 M (system VII) and in 0.5 M (system VIII) salt solutions were considered to check the possibility of the existence of dimers. System IX contains 18 molecules of trans-Cd(Phen)2 in water, and system X contains 36 molecules of trans-Cd(Phen)2 in a 1 M salt solution. System X was obtained from system IX via the addition of Na^+^ and Cl^−^ ions.

It can be noted that systems with water and without salt contain the necessary amount of Cl^−^ ions for electrical neutrality (systems I, III, V, VI, and IX).

Also, the systems of DNA with Cd(Phen)3 (system DI) or with Cd(Phen)2 in cis- and in trans-conformations (systems DII and DIII) in water were considered (see Table 2). The double-stranded DNA fragment contains 21 base pairs with the sequence GCCCAGCATTTCACCCAGATT and an overall charge of −42 (e). The DNA molecule stretches from one face of a simulation box to another, so with periodic boundary conditions, the DNA molecule becomes infinite and has no ends. This approach allows us to exclude the sticking of Cd-complexes to the ends of DNA fragments, which is outside our research focus.

A molecular dynamics simulation was carried out in the AKMD program [29] using the AMBER-14sb [30] forcefield and the SPC/E model for water; compatible parameters for Na^+^ and Cl^−^ developed by Dang [31] and Li [32] were used. The difference in potentials (Dang or Li) does not affect the simulation result. Both Cd(Phen)3 and Cd(Phen)2 have a charge of +2e. A time step of 1 fs was used. The simulation was carried out in the NTV ensemble with a Nosé–Hoover thermostat [33]. The length of the simulation box for the systems containing DNA was fixed to 70.98 Å, which coincides with the length of the DNA fragment of 21 base pairs. The width and height of the simulation box were adjusted so that the average pressure in the system was approximately 1 atm. The temperature was kept at 25 °C. The electrostatic interactions were treated with the Ewald method [34]. The lengths of the molecular bonds were maintained with the SHAKE algorithm [35].

## 4. Conclusions

Based on all data obtained, it can be argued that cadmium compounds Cd(Phen)3, Cd(Phen)2, and 2Cd(Phen)4 interact with DNA. There are many similarities in their interactions with DNA. This is especially true for the spectral changes recorded during the formation of complexes. This indicates the similarity in the interactions of the phenanthroline ligands of the compounds with DNA. The hypochromic effect in the absorption spectra of the compounds in the complexes with DNA points to stacking interactions involving 1,10 phenanthroline. This may be both a consequence of intercalation and the result of the formation of phenanthroline dimers upon external binding of compounds to DNA. An external binding is preferable for compound Cd(Phen)3. The location of hydrophobic ligands on the outside of the DNA helix provokes the formation of intermolecular DNA-DNA linkages and the appearance of aggregates in the solution. This is confirmed by DLS data. Without excluding the possibility of external binding of compounds Cd(Phen)2 and 2Cd(Phen)4 to DNA, we still believe that, for these compounds, the main type of binding, in addition to electrostatic, is intercalation. In this case, phenanthroline remains in the coordination sphere of the complex ion.

The possibility of the formation of the dimers of the Cd(Phen)2 compound with the stacking of 1,10-phenanthrolines during the external binding was confirmed by computer simulations for systems containing salt. Therefore, the stacking interaction of phenanthroline ligands is very likely after the compensation of the charge of the complex ions during their binding to DNA phosphates. Thus, the external binding of Cd(Phen)2 to DNA is also possible. However, the termination of the growth of the viscosity of DNA solutions at r > 1 may indicate the filling of possible binding sites on DNA for Cd(Phen)2. This is different from the external binding of Cd(Phen)3 to DNA, which induces the emergence of aggregates. Finally, simulations showed that compound Cd(Phen)3 does not penetrate DNA grooves, unlike compound Cd(Phen)2, which is a necessary step for the Intercalation.

We can assume the formation of a coordination bond between cadmium and DNA only for the complexes of DNA with 2Cd(Phen)4. Indeed, the difference in the binding of compounds to DNA, which appears in viscometry studies, especially in experiments with different dilutions of complexes, indicates that the strong binding with the formation of the coordination bonds is realized only for the binuclear compound. In this case, most likely, the intercalation of 1,10 phenanthroline also occurs. We believe that for the mononuclear compound Cd(Phen)2, the intercalation model is suitable but without coordination of the cadmium ion to DNA. Note that the binding constant can be determined only for equilibrium binding. This definition is incorrect for complexes with coordination bonds. However, most likely, at least two types of binding are realized for a binuclear complex, without considering the electrostatic interaction of cadmium ions with DNA phosphate groups. When a binuclear cadmium compound binds to DNA, only one complex ion participates in the complex formation. It may be that the coordination of the Cd ion to DNA in a major groove is accompanied by the simultaneous insertion of 1,10-phenanthroline between the bases. It is also possible that, on the contrary, coordination binding occurs in the major groove of DNA, and the intercalation of 1,10-phenanthroline also occurs from the minor or major grooves.

At high salt concentrations, the DNA interaction with cadmium compounds gets weaker. Under these conditions, a tendency toward DNA aggregation appears. In DNA solutions the hydrophobic 1,10-phenanthrolines from the coordination sphere of cadmium provoke the formation of intermolecular DNA-DNA bonds.

Computer simulation data confirm the formation of DNA complexes with compounds Cd(Phen)3 and Cd(Phen)2 with the penetration of complex ions into the major (for Cd(Phen)3) and minor (for Cd(Phen)2) grooves of DNA. Further research in this area is required.

The main goal of the research was to determine the molecular mechanism of the DNA interaction with cadmium compounds. A heterocyclic 1,10-phenanthroline exerts in vitro antimicrobial activity against a broad spectrum of bacteria. Metal coordination compounds also exhibit biological activity. The combination of the properties of the components of metal coordination compounds makes it possible to enhance the therapeutic effect. As shown in our study, the location of the ligands in the coordination sphere of the ion determines the interaction of the cadmium compound with DNA. The antimicrobial activity of phenanthroline can be significantly modulated by modifying the structure of the compounds. The cadmium compounds with two 1,10-phenanthroline ligands in the coordination sphere of an ion provide a greater opportunity to form biologically significant complexes via intercalation. At the same time, phenanthroline can manifest itself as an intercalator, and simultaneously, cadmium can coordinate with DNA bases only in binuclear cadmium compounds.

## Figures and Tables

**Table 1:** The simulated systems of Cd(Phen)3 and Cd(Phen)2 in water and in salted solutions.

	I	II	III	IV	V	VI	VII	VIII	IX	X
Cd(Phen)3	9	9	-	-	-	-	-	-	-	-
cis-Cd(Phen)2	-	-	9	9	9	36 (in the form of dimers)	36 (in the form of dimers)	36 (in the form of dimers)	-	-
trans-Cd(Phen)2	-	-	-	-	-	-	-	-	18	36
Cl^−^	18	641	18	633	18	72	173	413	36	683
Na^+^	-	623	-	615	-	-	101	341	-	611

**Table 2:** The simulated systems of DNA with Cd(Phen)3 or Cd(Phen)2.

	DI	DII	DIII
Cd(Phen)3	8	-	-
cis-Cd(Phen)2	-	8	-
trans-Cd(Phen)2	-	-	8
Na^+^	26	26	26
H_2_O	12,210	12,366	12,360
box size, Å	70.98 × 73 × 73	70.98 × 73 × 73	70.98 × 73 × 73
Time, ns	43	15	38

## Data Availability

All experimental data and calculations are contained in the archive of the Laboratory of Molecular Biophysics of St. Petersburg State University.

## References

[1] Rosenberg B., Van Camp L., Krigas T. (1965). Inhibition of cell division in Escherichia coli by electrolysis products from a platinum electrode. Nature.

[2] Rosenberg B. (1971). Some Biological effects of platinum compounds. Platin. Met. Rev..

[3] Alassadi S., Pisani M.J., Wheate N.J. (2022). A Chemical perspective on the clinical use of platinum-based anticancer drugs. Dalton Trans..

[4] Gandin V., Hoeschele J.D., Margiotta N. (2023). Special Issue “Cisplatin in Cancer Therapy: Molecular Mechanisms of Action 3.0”. Int. J. Mol. Sci..

[5] Marverti G., Gozzi G., Lauriola A., Ponterini G., Belluti S., Imbriano C., Costi M.P., D’Arca D. (2019). The 1,10-phenanthroline ligand enhances the antiproliferative activity of DNA-intercalating thiourea-Pd(II) and Pt(II) complexes against cisplatin-sensitive and -Resistant human ovarian cancer cell lines. Int. J. Mol. Sci..

[6] Janjić G.V., Petrović P., Ninković D., Zarić S.D. (2011). Geometries of stacking interactions between phenanthroline ligands in crystal structures of square- planar metal complexes. J. Mol. Model..

[7] Oguadinma P.O., Rodrigue-Witchel A., Reber C., Schaper F. (2010). Intramolecular π-stacking in copper(I) diketiminate phenanthroline complexes. Dalton Trans..

[8] Nisbet M.L., Wang Y., Poeppelmeier K.R. (2021). Symmetry-dependent intermolecular π-π stacking directed by hydrogen bonding in racemic copper-phenanthroline compounds. Cryst. Growth Des..

[9] Barton J.K. (1983). Tris (phenanthroline) metal complexes: Probes for DNA helicity. J. Biomol. Struct. Dyn..

[10] Williams L.D. (1988). Specific binding of o -phenanthroline at a DNA structural lesion. Nucl. Acid Res..

[11] Carter M.T., Rodriguez M., Bard A.J. (1989). Voltammetric studies of the interaction of metal chelates with DNA. 2. Tris-chelated complexes of cobalt(III) and iron(II) with 1,l0-phenanthroline and 2,2′-bipyridine. J. Am. Chem. Soc..

[12] Takezawa Y., Kanemaru D., Kudoa N., Shionoya M. (2023). Phenanthroline-modified DNA three-way junction structures stabilized by interstrand 3:1 metal complexation. Dalton Trans..

[13] Sánchez-González Á., Bandeira N.A.G., de Luzuriaga I.O., Martins F.F., Elleuchi S., Jarraya K., Lanuza J., Lopez X., Calhorda M.J., Gil A. (2021). New Insights on the interaction of phenanthroline based ligands and metal complexes and polyoxometalates with duplex DNA and G-quadruplexes. Molecules.

[14] Vanani A.R., Asadpour S., Aramesh-Boroujeni Z., Dehkordi M.M. (2023). Studying the interaction between the new neodymium (Nd) complex with the ligand of 1,10-phenanthroline with FS-DNA and BSA. Front. Chem..

[15] Demidov V.N., Kasyanenko N.A., Antonov V.S., Volkov I.L., Sokolov P.A., Pakhomova T.B., Simanova S.A. (2012). Reaction with DNA and pharmacologic activity of 1,10-phenanthroline and electron-rich 1,10-phenanthrocyanine complexes of d-elements. Rus. J. Gen. Chem..

[16] Kasyanenko N., Qiushi Z., Bakulev V., Osolodkov M., Sokolov P., Demidov V. (2017). DNA Binding with Acetate Bis(1,10-phenanthroline)silver(I) Monohydrate in a Solution and Metallization of Formed Structures. Polymers.

[17] Kasyanenko N.A., Tikhomirov A.R., Bakulev V.M., Demidov V.N., Chikhirzhina E.V., Moroshkina E.B. (2019). DNA Complexes with Cobalt(II) Phthalocyanine Disodium Disulfonate. ACS Omega.

[18] Luevano J., Damodaran C. (2014). A review of molecular events of cadmium-induced carcinogenesis. J. Environ. Pathol. Toxicol. Oncol..

[19] Genchi G., Stefania M.S., Lauria G., Carocci A., Catalano A. (2020). The Effects of Cadmium Toxicity. Int. J. Environ. Res. Public Health.

[20] Hajdu B., Hunyadi E., Gyurcsi E. (2023). Interactions of an Artificial Zinc Finger Protein with Cd(II) and Hg(II): Competition and Metal and DNA Binding. Inorganics.

[21] Lerman L.S. (1961). Structural considerations in the interaction of DNA and acridines. J. Mol. Biol..

[22] Baguley B.C., Le Bret M. (1984). Quenching of DNA-ethidium fluorescence by amsacrine and other antitumor agents: A possible electron-transfer effect. Biochemistry.

[23] Reinert K.E. (1973). DNA stiffening and elongation caused by the binding of ethidium bromide. Biochim. Biophys. Acta (BBA)-Nucleic Acids Protein Synth..

[24] Miller K.J., Pycior J.F. (1979). Interaction of molecules with nucleic acids. II. Two pairs of families of intercalation sites, unwinding angles, and the neighbor-exclusion principle. Biopolymers.

[25] Fiel R.J., Howard J.C., Mark E.H., Gupta N.D. (1979). Interaction of DNA with a porphyrin ligand: Evidence for intercalation. Nucleic Acids Res..

[26] Demidov V.N. (2010). Electron-Rich 1,10-Phenanthrocyanine Complexes of d-Elements: Formation Patterns, Spectral Properties, Structural and Thermodynamic Similarity. Ph.D. Thesis.

[27] Wolfe A., Shimer G.H., Meehan T. (1987). Polycyclic aromatic hydrocarbons physically intercalate into duplex regions of denatured DNA. Biochemistry.

[28] Frisman E.V., Shchagina L.V., Vorobev V.I. (1965). A glass rotating viscometer. Biorheology.

[29] Komolkin A.V., Laaksonen A., Maliniak A. (1994). Molecular Dynamics Simulation of a Nematic Liquid Crystal. J. Chem. Phys..

[30] Maier J.A., Martinez C., Kasavajhala K., Wickstrom L., Hauser K.E., Simmerling C. (2015). ff14SB: Improving the Accuracy of Protein Side Chain and Backbone Parameters from ff99SB. J. Chem. Theory Comput..

[31] Dang L.X. (1995). Mechanism and Thermodynamics of Ion Selectivity in Aqueous Solutions of 18-Crown-6 Ether: A Molecular Dynamics Study. J. Am. Chem. Soc..

[32] Li P., Song L.F., Merz K.M. (2015). Systematic Parameterization of Monovalent Ions Employing the Nonbonded Model. J. Chem. Theory Comput..

[33] Hoover W.G. (1985). Canonical Dynamics—Equilibrium Phase- Space Distributions. Phys. Rev. A.

[34] Ewald P. (1921). The Calculation of Optical and Electrostatic Grid Potential. Ann. Phys..

[35] Ryckaert J.-P., Ciccotti G., Berendsen H.J.C. (1977). Numerical Integration of the Cartesian Equations of Motion of a System with Constraints: Molecular Dynamics of n-alkanes. J. Comput. Phys..

